# Genetic information and insurance

**DOI:** 10.1038/s44319-024-00229-z

**Published:** 2024-08-14

**Authors:** Frank Gannon

**Affiliations:** QIMR Berghofer Medical Research Institute, Brisbane, QLD Australia

**Keywords:** Economics, Law & Politics, Genetics, Gene Therapy & Genetic Disease, Molecular Biology of Disease

## Abstract

The use of genetic data for diagnosis increases the risk of genetic discrimination by insurance companies and could hinder progress in medicine.

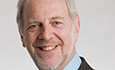

A friend of mine was recently diagnosed with cancer. It was devastating for her, in particular as two of her daughters have also been recovering from different cancers. But she has so far not acted to get the tumor or her germ-line cells sequenced as part of her diagnosis—perhaps because her family history suggests the existence of some inheritable genetic elements that could spell bad news not only for her children but also the next generation. That knowledge could be an emotional burden. But this may be just one reason: when I spoke to others, one of them pointed me to the possible problem of getting a life insurance at a reasonable cost once negative genetic data are available. This would be an example of Genetic Discrimination.

I wondered if this is an issue specific to Australia. The answers to emails to friends in the USA, Germany and the UK indicated that the problem of linkages between insurance and genetic information exists elsewhere and that each country addresses it differently. Further analysis of data collected by the Geneva Association revealed that only Austria, Belgium, Canada, Denmark, France, Ireland, Poland and Portugal prohibit the use of genetic information for insurance purposes (https://www.genevaassociation.org/sites/default/files/ga2017_globalageing_genetics_and_life_insurance_0.pdf). In Australia, Germany, The Netherlands, and Switzerland, the capacity of insurers to use genetic information to set premiums depends on the amount of insurance requested. Other countries have no legislation, only guidelines, moratoria or waivers that insurance companies can use at their discretion. In other words, Genetic Discrimination is practiced widely and in a variable manner depending on which country you live in.

Although life insurance is the most obvious insurance product to be controlled—or not—by national regulations, the issue extends to other types of cover. Many self-employed workers seek income protection or permanent disability insurance to cover for unforeseeable accidents or disability. Banks often require a life insurance as security for mortgages. More generally, many people have insurances to financially support their families in the event of their early demise. Insurances, including travel, are a common and popular product just in case something goes wrong. But if you live in a country without legislation that either bans or at least defines the use of genetic information by insurance companies, the day you acquire genetic data leaves a trail that may affect your children’s ability to get insured. Your genes are a “pre-existing condition” and your refusal to disclose genetic data if it exists may be used against you when you make a claim. Thus, Genetic Discrimination can really hurt many healthy people financially.

In a medical setting, the need for greater awareness of insurance implications should come when conversations with a clinician points to the benefit of acquiring genetic data—even if is both difficult and insensitive to discuss the issue at that particular moment. However, if it is not raised, the patients may eventually feel that they were not adequately informed of the longer-term consequences. Some genetic counseling practices therefore advise patients to avoid obtaining genetic data until they are over eighteen and have signed a life insurance contract.

Still, there are other ways by which genetic data can potentially find its way into the hands of an insurance company. Healthy volunteers for research or clinical trials, who, as part of the trial, have their genome sequenced, have to be made aware of potential inquiries from insurers in the future. Customers who send samples to direct-to-consumer genetic testing companies, such as 23andMe, can ask for FDA-approved reports about the risk of developing specific diseases. The increasing importance of Polygenic Risk Scores will further add to the narrative that everyone carries a higher-than-average risk for developing some diseases during their lifetime. In the future, algorithms will convert that score into a premium on an insurance policy that has asked for any genetic information known to the applicant. Genetic data are increasingly being collected and used for a wide variety of reasons and, as this trend is accelerating, the implications become a topic for all citizens.

With the push towards precision medicine—the right treatment with the right dose at the right time—collecting genetic information will become a standard part of diagnosis. However, the willingness to get health benefits from such information may be undermined if the genetic data becomes a financial liability. It is not clear how insurance companies will factor in the many uncertainties of interpretation into their costing of an insurance policy, given that even experts struggle with distinguishing a mutation of clinical relevance from one that is not. Neither can we know how insurance companies will calculate the effect of lifestyle and resulting variations in epigenetic modifications on some genetic irregularities. Nor do we know if or when the whole genome sequence will be a routine requirement by insurers and re-insurers. If it is, or even portions of it, what are the rights of privacy for the insured and how should the transfer of data across country borders be handled? Many related problems are brewing.

From the perspective of the medical researcher or genetic counselor, the argument to ban the use of genetic data, as has happened in Canada for instance, is a wise move and would leave open the path to better treatments. The likely response from insurance companies is to increase premiums on all policies to exert pressure on governments which may delay new laws that would prevent Genetic Discrimination. However, there is a flaw in that logical basis of the demand should companies choose to float that threat. Before genetic sequencing, they had to rely on other indicators such as disease in first-degree family members, the general health of the individual looking for life insurance and lifestyle factors. Omitting the rich but confusing genetic data will not interfere with their normal and generally profitable business. Banning its use by insurance companies will therefore allow progress in medical research and give greater benefits to society.

## Supplementary information

Peer Review File

