# Supplementary material for: Genetic information and insurance
Source: EMBO Rep. 2024 Aug 14;25(9):3741–2. doi: 10.1038/s44319-024-00229-z (PMC11387390; doi:10.1038/s44319-024-00229-z)
Supplement: Supplementary file 1 — Peer Review File [file 44319_2024_229_MOESM1_ESM.pdf]

EMBO reports

Genetic information and Insurance

Frank Gannon

Corresponding author(s): Frank Gannon (FGannon@qimrberghofer.edu.au)

Review Timeline:

Submission Date:	30th Jul 24
Accepted:	7th Aug 24

Editor: Holger Breithaupt

Transaction Report: This article was not peer reviewed.
